# Fronto-temporoparietal connectivity and self-awareness in 18-month-olds: A resting state fNIRS study

**DOI:** 10.1016/j.dcn.2019.100676

**Published:** 2019-06-22

**Authors:** Chiara Bulgarelli, Anna Blasi, Carina C.J.M. de Klerk, John E. Richards, Antonia Hamilton, Victoria Southgate

**Affiliations:** a Centre for Brain and Cognitive Development, Birkbeck College, University of London, UK; b Department of Medical Physics and Bioengineering, University College London, UK; c Department of Psychology, University of Essex, UK; d University of South Carolina, Institute for Mind and Brain, Department of Psychology, United States; e Institute of Cognitive Neuroscience, University College London, UK; f Department of Psychology, University of Copenhagen, Denmark

**Keywords:** Self-awareness, fNIRS, Functional connectivity, Resting-state, Toddler development, Default mode network

## Abstract

How and when a concept of the ‘self’ emerges has been the topic of much interest in developmental psychology. Self-awareness has been proposed to emerge at around 18 months, when toddlers start to show evidence of physical self-recognition. However, to what extent physical self-recognition is a valid indicator of being able to think about oneself, is debated. Research in adult cognitive neuroscience has suggested that a common network of brain regions called Default Mode Network (DMN), including the temporo-parietal junction (TPJ) and the medial prefrontal cortex (mPFC), is recruited when we are reflecting on the self. We hypothesized that if mirror self-recognition involves self-awareness, toddlers who exhibit mirror self-recognition might show increased functional connectivity between frontal and temporoparietal regions of the brain, relative to those toddlers who do not yet show mirror self-recognition. Using fNIRS, we collected resting-state data from 18 Recognizers and 22 Non-Recognizers at 18 months of age. We found significantly stronger fronto-temporoparietal connectivity in Recognizers compared to Non-Recognizers, a finding which might support the hypothesized relationship between mirror-self recognition and self-awareness in infancy.

## Highlights

• There is no agreement on the significance of mirror self-recognition test in infancy.
• In adults, the DMN has been associated with abstract self-processing.
• Recognisers showed greater fronto-temporoparietal connectivity than Non-Recognisers.
• Self-recognition may reflect a genuine advance in toddlers’ self-awareness.

## 1 Introduction

The emergence of a child’s sense of self has long been a topic of interest in psychology, but research has been limited because the sense of self is difficult to operationalize empirically. There appears to be a consensus that we are born with some ‘minimal’ sense of self that allows us to interact with the environment (Zahavi, 2017), and empirical work suggests that young infants have some rudimentary bodily self-perception abilities (Filippetti et al., 2015). Many scholars have, however, distinguished between different levels of self-awareness (Damasio, 1999; James, 1890; Neisser, 1988; Panksepp, 1998; Rochat, 2010) and within the literature, there is an intuitive assumption of a distinction between physical self-awareness and psychological or cognitive self-awareness (Dennett, 1989; Gillihan and Farah, 2005; Howe et al., 1993). Given that there are likely to be different levels on which one could be said to be self-aware, it is still unclear how we should characterize the kind of self-awareness that is indexed with classic tests of self-awareness in infancy (Bard et al., 2006; Gadlin and Ingle, 1975; Suddendorf, 2003). The still dominant means of assessing self-perception in infancy is the mirror self-recognition (MSR) task (Amsterdam, 1972; Rochat, 2003). In this task, toddlers’ behaviour in front of the mirror is assessed after a red mark has been covertly placed on their cheek. If the toddler touches the mark on their face, this is taken as an index of physical self-recognition, suggesting that the toddler identified something unusual in their own appearance. While younger children placed in front of a mirror appear to perceive their specular image as an extension of the environment, from around 18 months of age, toddlers begin to show evidence of self-recognition, suggesting that at around this age, they understand that what they see in the mirror is themselves (Rochat, 2003). While, strictly speaking, the MSR task measures physical self-recognition, many have argued that recognizing oneself in the mirror is an ability that reflects a broader conceptualization of the self, i.e. the capability to think about oneself as a particular individual with specific physical and psychological features (Gallup et al., 2016; Suddendorf and Butler, 2013), including elements of psychological self-awareness. For example, success on the MSR test is associated with empathy (Bischof-Köhler, 2012), as well as personal pronoun use and pretend play (Lewis and Ramsay, 2004), each of which has been argued to require self-awareness (Brandl, 2016). Self-recognition in the mirror has also been associated with the use of symbols, with the mirror being considered a symbol of the representations of one’s own body (Savanah, 2013). Furthermore, there is evidence that self-recognition in the mirror is associated with memory (Harley and Reese, 1999; Howe et al., 1993; Prudhomme, 2005). In one of these studies, researchers found that infants who were classified as recognizers on the MSR exhibited better memory for the location of a hidden object 12 months later, a finding that is consistent with the hypothesized relationship between cognitive self-representation and memory organization (Howe et al., 1993) and consistent with the hypothesis that MSR indexes a cognitive self-awareness. Nevertheless, despite being the dominant measure of emerging self-awareness, there is still no general acceptance of the claim that the MSR test reflects a developing self-awareness (for critics see Heyes, 1994; Mitchell, 1993a,b), and some have argued that it may instead reflect an understanding of the properties of mirrors (Loveland, 1986), or differences in the extent to which toddlers are either able or motivated to touch the mark (Asendorpf et al., 1996).

Studying the development of self-awareness in toddlers is challenging as they cannot report on how they process self-related stimuli. Therefore, our current knowledge of early self-awareness is limited, while much work in adult cognitive neuroscience has already made significant progress in identifying the neural underpinnings of self-related processing. Specifically, a network of brain regions which is activated during passive rest (i.e. resting-state) in the low‐frequency range (<0.1 Hz), appears to be recruited during self-related processing (Raichle, 2015). This so-called Default Mode Network (DMN), which overlaps considerably with the social brain network (Mars et al., 2012), is composed of the medial prefrontal cortex (mPFC), the precuneus, the posterior and anterior cingulate cortex, the inferior parietal lobe (IPL), the medial temporal lobe and the temporoparietal junction (TPJ) (Davey et al., 2016; Greicius et al., 2003; Harrison et al., 2008; Mars et al., 2012; Molnar-Szakacs and Uddin, 2013; Raichle, 2015; Schilbach et al., 2008; Sporns, 2010). The DMN is thought to play a pivotal role in several introspective and adaptive mental activities, such as autobiographical memory (Philippi et al., 2014; Yang et al., 2013), theory of mind and mentalizing (Li et al., 2014; Mars et al., 2012), and planning and envisioning future events (Østby et al., 2012; Xu et al., 2016). Self-referential mental processing is the common feature of most of the processes which elicit DMN engagement, suggesting that this network is our ‘intrinsic system’ for dealing with self-related processing (Davey et al., 2016; Golland et al., 2008; Molnar-Szakacs and Uddin, 2013; Raichle, 2015; Sporns, 2010). Consistent with this idea, recent fMRI studies have shown the engagement of core areas of the DMN in self-processing tasks in adults (Davey et al., 2016; Kaplan et al., 2008; Kelley et al., 2002; Kircher et al., 2000; Uddin et al., 2005). The fact that the activity elicited by self-processing tasks is remarkably similar to the activation of the DMN during rest, led to the hypothesis that during quiet rest there is a shift from perceiving the external world to internal modes of cognition (Buckner and Carroll, 2007). This has been empirically supported by imaging studies demonstrating that DMN activity at rest is positively correlated with participants’ reports of mind-wandering and self-related thoughts (Mason et al., 2007; McKiernan et al., 2006). Importantly, the DMN appears to be primarily involved in psychological self-processing and less so in physical self-recognition (Northoff et al., 2006).

To date, we know very little about the neural underpinnings of self-awareness in the developing brain. However, given the debate surrounding the validity of the MSR task as an indicator of self-awareness beyond physical self-recognition (Suddendorf and Butler, 2013), investigating the relationship between the brain regions implicated in adult self-awareness and self-recognition in the mirror in toddlers, could inform this debate. Specifically, if it is the case that mirror self-recognition reflects self-awareness beyond mere physical self-recognition, as is suggested by reported associations between MSR and empathy and personal pronoun use (Bischof-Köhler, 2012; Lewis and Ramsay, 2004), then we might expect those children who pass the MSR test to have higher resting-state connectivity within their default mode network. Furthermore, if the integrity of this network is associated with successful mirror self-recognition, it would suggest that mirror self-recognition is unlikely to be an artefact of motivation or understanding the properties of mirrors. To our knowledge, the only study that investigated the neural substrates of the developing sense of self was performed with structural MRI on 15 toddlers from 15 to 30 months of age, focusing on the relationship between brain maturation and self-representation (as indexed by the MSR task, other-directed pretended play and use of personal pronouns) (Lewis and Carmody, 2008). This study found that brain maturation in TPJ specifically was associated with self-recognition in toddlers, suggesting a role for this area in the emergence of self-awareness. Interestingly, a positive relationship between self-recognition and empathy (Bischof-Köhler, 2012) and between self-recognition and brain maturation in TPJ (Lewis and Carmody, 2008) exist even after controlling for age, suggesting that emerging self-awareness might not be explained by a general maturation process. There are also several studies documenting the maturation of the DMN during the first years of life (e.g. see Emberson et al., 2015; Fransson et al., 2011; Homae et al., 2011), which suggest that the DMN is adult-like by the middle of the second year of life (Gao et al., 2017), consistent with success on the MSR at around this age. However, to date, we do not know if self-awareness might be related to the maturity of the DMN. The aim of the current study was to test this hypothesis. Specifically, we investigated whether resting-state functional connectivity (RSFC) between frontal and temporoparietal brain regions was greater in 18-month-old toddlers who exhibited mirror self-recognition compared with those who did not.

Functional near-infrared spectroscopy (fNIRS) has emerged as a viable method for investigating RSFC in toddlers. fNIRS is a non-invasive neuroimaging method, which allows the measurement of changes in concentration of oxy-haemoglobin (HbO_2_) and deoxy-haemoglobin (HHb), indexing brain activation (Elwell, 1995; Hoshi, 2016). Unlike fMRI, fNIRS has been widely used in task-related studies with awake infants (Ferrari and Quaresima, 2012; Lloyd-Fox et al., 2010), and consequently it is a method that can be used for acquiring resting-state recordings under similar conditions to those used in studies investigating DMN involvement in self-related processing in adults. Additionally, compared to other infant-friendly neuroimaging methods such as electroencephalography, fNIRS offers clear advantages for assessing functional connectivity because the light intensity measured at the scalp level can be spatially localized with higher resolution. Moreover, fNIRS also provides a relatively high temporal resolution of the spontaneous fluctuations of the haemodynamic response, a valuable feature in connectivity analyses (Lee et al., 2013). Indeed, previous adult studies have used fNIRS to assess RSFC, suggesting it is a promising tool for this purpose (Lu et al., 2010; Mesquita et al., 2010). However, due to the inherent properties of fNIRS, its use is limited to the outer layers of the cortex. Therefore, in this study we measured connectivity between frontal, temporal, and parietal brain areas, which we will refer to as fronto-temporoparietal connectivity, as a proxy for the DMN. The approach of studying some portions of the DMN as a proxy for this network has also been adopted in studies with adults, focusing in particular on the mPFC (Durantin et al., 2015; Liang et al., 2016; Sasai et al., 2012) and on the parietal lobes (Rosenbaum et al., 2017; Sasai et al., 2012).

In the current study, we employed fNIRS during a state of quiet restfulness to investigate the relationship between fronto-temporoparietal functional connectivity, as a putative index of DMN activity, and self-awareness as measured by the MSR task. Based on previous literature suggesting that mirror self-recognition reflects a cognitive conceptualization of the self (e.g. Howe et al., 1993), and adult data implicating the DMN in this process, we hypothesized that fronto-temporoparietal connectivity, as measured by resting-state fNIRS, would be greater in toddlers capable of self-recognition than in those who do not show evidence of MSR.

## 2 Method

### 2.1 Participants

We acquired resting-state data from 43 18-month-olds (23 males, age mean ± SD = 553.11 ± 12.17 days). An additional 52 toddlers were excluded because: (i) their dataset did not reach a minimum of 100 s of recording after behavioural coding (28 toddlers); (ii) they refused to wear the fNIRS hat or the fNIRS headgear/hat was not well fitted (18 toddlers); (iii) more than 30% of the channels had to be excluded due to poor light intensity readings (6 toddlers). For more details about behavioural coding and mean intensity readings per channel, see Section 2.6.

All included toddlers were born full- term, healthy and with normal birth weight. Written informed consent was obtained from the toddler’s caregiver prior to the start of the experiment.

### 2.2 MSR and coding scheme

Prior to the fNIRS resting-state acquisition, self-awareness was assessed with the MSR task (Amsterdam, 1972). This task took place in a room with a mirror positioned against one of the walls. One experimenter focused on recording the testing session, she entered the testing room first and hid behind the curtains to avoid interfering with the testing session. A second experimenter first engaged the toddler in a warm-up play session, and then redirected the toddler’s attention to the mirror. Once the toddler had visually fixated the mirror image of his/her face at least three times, the experimenter covertly applied a red dot with lipstick on his/her cheek, while pretending to wipe the toddler’s nose. After this, the experimenter again engaged the toddler in front of the mirror, making sure that the toddler looked at him/herself at least three times. The experimenter prompted the toddler to look at the mirror by saying ‘Look there’ whenever necessary, but the only prompt for self-recognition that was used was the question "Who is that?", for a maximum of five times. This is a similar procedure used by other toddler studies (Asendorpf and Baudonnière, 1994; Asendorpf et al., 1996; Kristen-Antonow et al., 2015; Lewis and Carmody, 2008; Nielsen et al., 2003; Zmyj et al., 2013). The experimenter used bubbles to engage the toddler in playing, both before and after the red mark was placed (to avoid any differences between the two parts of the test). The caregiver was present in the room for the entire testing session, but was asked not to direct the toddler’s attention to his/her image in the mirror and to stay outside of the visual field reflected in the mirror, to prevent the toddler from seeing the caregiver’s reflected image as a cue for self-recognition. The experimenter also remained outside the mirror field of view.

Two experimenters independently classified the toddlers as ‘Recognisers’, ‘Ambiguous’, or ‘Non-Recognisers’ based on their behaviours in front of the mirror after the red mark was placed, and they agreed in 96% of the cases. Discrepancies were discussed until agreement was reached. Participants were defined as ‘Recognisers’ if they touched the cheek with the red mark, the nose or the other cheek. They were classified as ‘Ambiguous’ if they said their name while looking at themselves in the mirror but did not touch their face. All other behaviours fell in the Non-Recognisers category.

### 2.3 fNIRS recording and array configurations

fNIRS data was recorded using the UCL-NIRS topography system, which uses two continuous wavelengths of near-infrared light (770 nm and 850 nm) to detect changes in HbO_2_ and HHb concentration (Everdell et al., 2005). Sampling rate of data acquisition was 10 Hz.

**Fig. 1:**
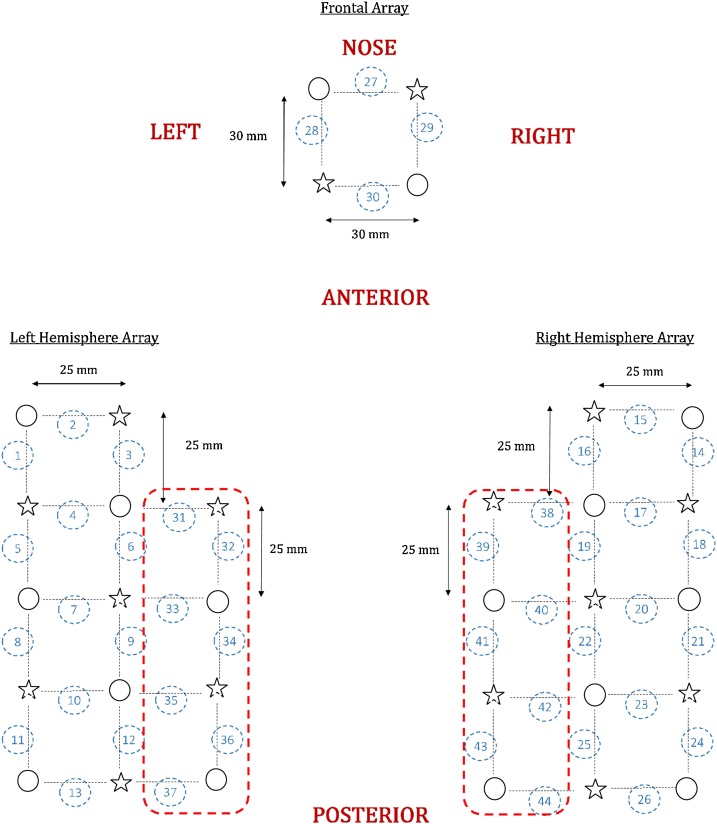
Representation of the fNIRS arrays. Sources are marked with stars, detectors are marked with circles, channels are marked with black dotted lines and numbered with blue circles. The red dotted lines highlight the additional rows of optodes that added 7 channels per hemisphere (results from the 44-channel configuration are presented in the Supplementary materials).

The toddlers were fitted with a custom-made NIRS headgear embedded in a flexible cap (EasyCap) covering the temporoparietal and frontal areas^1^ bilaterally in two very similar array designs. The first array design included 12 sources and 12 detectors to create a total of 30 measuring points (channels) and it was used to test 20 out of the 43 participants; the second design included 16 sources and 16 detectors that defined a total of 44 channels. The 44-channel configuration was an extension of the 30-channel configuration and included two additional rows of optodes that added 7 channels per hemisphere, in a superior location to the two existing lateral arrays (see Fig. 1). This allowed us to improve detection of the spontaneous fluctuation over the temporoparietal region, a core area of interest for this study, and it was used to test 23 out of the 43 participants. Both configurations shared the design and the location of the channels covering frontal, inferior frontal and temporal regions (30 channels out of 44). The reduced sample size of the participants tested with the 44-channel configuration did not provide enough statistical reliability for the analysis performed on the extended configuration. Therefore, in this study we included results from the 30-channel configuration only, while results from the 44-channel configuration are presented in the Supplementary materials.

**Fig. 2:**
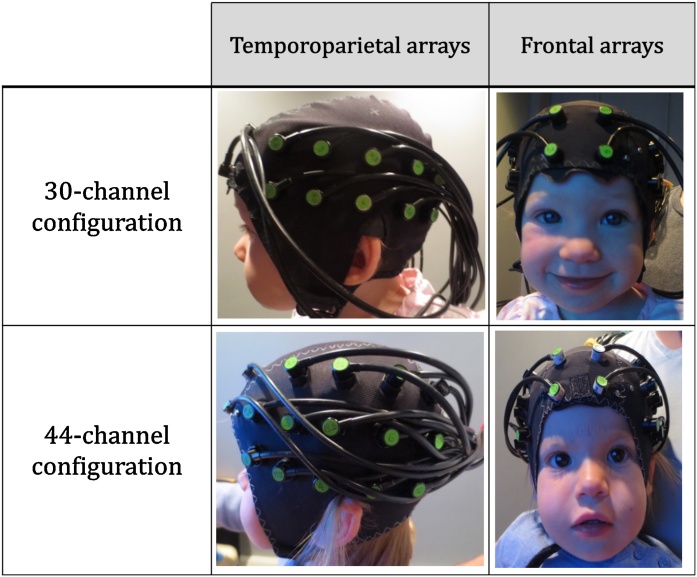
Pictures of the toddlers wearing the fNIRS cap. The first row represents the cap with the 30-channel configuration, and the second row the cap with the 44-channel configuration.

The EasyCap was made of soft black fabric, it was placed so that the third lower optode of the temporal array was centred above the pre-auricular point and that the two lower optodes of the frontal array centred over the nasion. Two differently sized caps (48 cm and 50 cm of circumference) were used to take into account variations in the toddlers’ head circumferences.^2^ Source-detector (S-D) separation was about 30 mm over the frontal lobe and 25 mm over the temporoparietal lobe. Given that the cortex is approximately 0.75 cm from the skin surface (Glenn, 2010) and based on studies on the transportation of near-infrared light through brain tissue, these selected source-detector separations were predicted to penetrate up to a depth of approximately 12.5–15 mm from the skin surface, allowing measurement of both the gyri and parts of the sulci near the surface of the cortex (Lloyd-Fox et al., 2010). S-D separation increased slightly due to the stretch of the cap on the head and also due to re-scaling based on the cap size. Table 1 lists information about S-D separation and number of toddlers included in the analysis who were tested with each cap size. Fig. 2 shows an example of toddlers wearing the two headgear configurations.

**Table 1:** S-D separation and number of participants tested for each cap size.

cap size	S-d temporoparietal lobe	S-d frontal lobe	Number of participants
48 cm	25 mm	30 mm	28/43
50 cm	26 mm	31 mm	15/43

### 2.4 Resting-state data acquisition

**Fig. 3:**
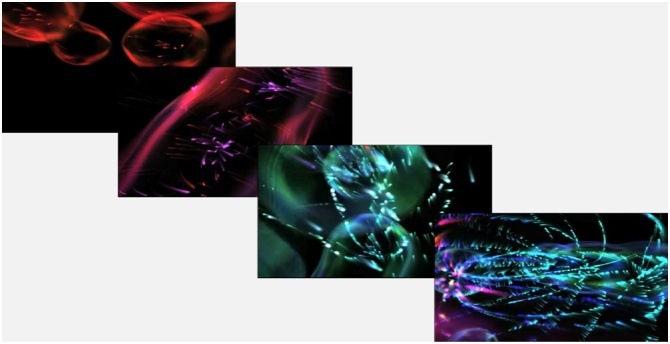
Still frames from the screensaver-like video shown during the resting-state acquisition.

The resting-state acquisition took place in a dimly lit and sound attenuated room, with the toddler sitting on their parent’s lap at approximately 90 cm from a 117 cm plasma screen. To keep the participants awake and as quiet as possible, we showed them a screensaver-like video with colourful bubbles accompanied by relaxing music (Fig. 3). The parent was asked not to talk during the experiment to avoid brain activation not associated with the spontaneous fluctuations during a state of quiet restfulness. If the parent talked to redirect the toddler’s attention to the screen or in case of fussiness or distraction, we excluded the corresponding section of data from the recording (see Section 2.6 for more details).

In fMRI resting-state studies, adult participants are typically asked not to think about anything in particular. However, recent studies have shown that the use of non-social movies or videos helps to keep participants awake, increases compliance, and helps avoid social or emotional thoughts during mind-wandering^3^ (Anderson et al., 2011; Cantlon and Li, 2013; Conroy et al., 2013; Sabuncu et al., 2010). Likewise, previous studies have used non-social videos to acquire resting-state with fMRI data in awake children (Müller et al., 2015; Vanderwal et al., 2015; Xiao et al., 2016). In adults, consistency within participants has been found between resting-state data acquired in a stimulus-free context and data acquired during observation of non-social videos, suggesting that observing such videos does not influence estimates of resting state connectivity significantly (Finn et al., 2017; Vanderwal et al., 2015).

### 2.5 Co-registration of the fNIRS array

**Fig. 4:**
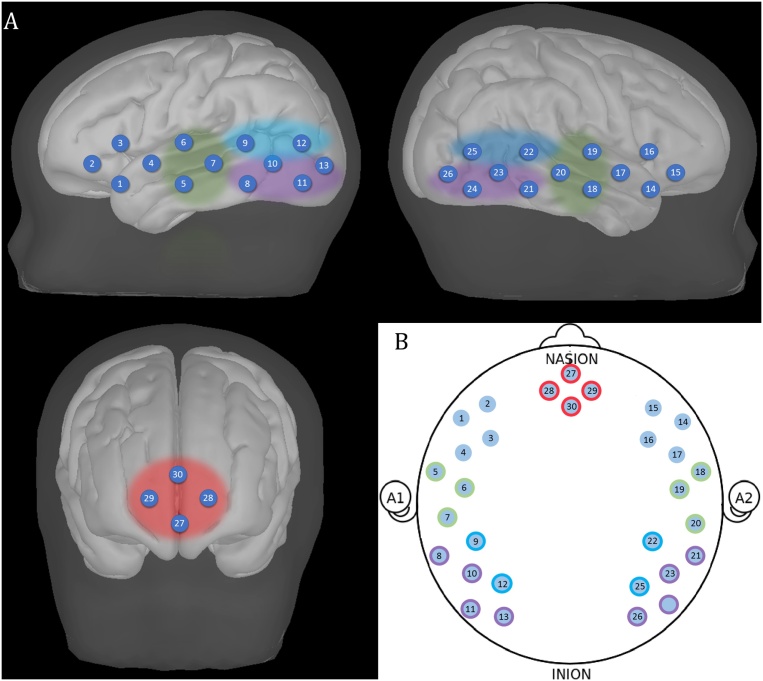
A, Representation of the channels on a 2-year-old structural template. B, Schematic representation of the channels. ROIs are highlighted: red represents mPFC, green represents STG, purple represents middle/posterior temporal gyrus, blue represents TPJ.

After the acquisition of the resting-state data, we logged the location of fNIRS array using the Polhemus Digitising System (http://polhemus.com/scanning-digitizing/digitizing-products/), if the participant was still compliant. We registered five reference points (nasion, inion, right ear, left ear, Cz^4^) and the location of the fNIRS optodes. This procedure allowed to co-register the fNIRS array on MRI structural scans, in order to infer more precisely the channels location. We selected the 10 best digitized recordings, based on the accuracy of the points marked in space compared to the optode locations in the pictures of the participant wearing the fNIRS cap that were taken after the recording (one from the front and two from the sides). For each of these recordings, a structural MRI of an toddler close in age with a similar head shape and size - based on head measurements collected before the testing session – was selected from the Neurodevelopmental MRI Database of the University of South Carolina (https://jerlab.sc.edu/projects/neurodevelopmental-mri-database/). The 10 fNIRS-MRI co-registrations were averaged together to estimate the location of the brain regions covered by the fNIRS array. The photon migration simulation was calculated for each channel using MCX (Fang and Boas, 2009), which estimates the paths of the photons from the source to the detector through the cortex. A cut-off of 25% of the voxels surrounding the spatial projection point was used to determine the anatomical label for each channel. Table 2 lists the anatomical labels (LPBA40 atlas) and the region of interest (ROI) associated to each channel belonging to the array design described in Section 2.3. Fig. 4 provides a graphical representation of the brain areas covered by the fNIRS array.

**Table 2:** Co-registration of each channel of the fNIRS array. The table shows anatomical labels (LPBA40 atlas) associated to each channel. Channels in bold are over the frontal cortex, channels marked with * are over the temporoparietal regions.

Channel	LPBA40 atlas	ROI	Channel	LPBA40 atlas	ROI
1	Inferior frontal gyrus, Superior temporal gyrus	Left IFG	16	Inferior frontal gyrus	Right IFG
2	Inferior frontal gyrus	Left IFG	17	Precentral gyrus	
3	Inferior frontal gyrus, Precentral gyrus	Left IFG	18*	Middle temporal gyrus, Superior temporal gyrus	Right STG
4	Precentral gyrus, Superior temporal gyrus		19*	Supramarginal gyrus	Right STG
5*	Middle temporal gyrus, Superior temporal gyrus	Left STG	20*	Middle temporal gyrus, Superior temporal gyrus	Right STG
6*	Postcentral gyrus	Left STG	21*	Middle temporal gyrus	Right PTG
7*	Middle temporal gyrus, Superior temporal gyrus	Left STG	22*	Supramarginal gyrus	Right TPJ
8*	Inferior temporal gyrus, Middle temporal gyrus	Left PTG	23*	Middle temporal gyrus	Right PTG
9*	Supramarginal gyrus	Left TPJ	24*	Middle temporal gyrus Inferior temporal gyrus	Right PTG
10*	Middle temporal gyrus	Left PTG	25*	Angular gyrus	Right TPJ
11*	Inferior temporal gyrus, Middle occipital gyrus	Left PTG	26*	Angular gyrus, Middle occipital gyrus	Right PTG
12*	Angular gyrus, Middle occipital gyrus	Left TPJ	**27**	Middle frontal gyrus, Superior frontal gyrus	mPFC
13*	Angular gyrus, Middle occipital gyrus	Left PTG	**28**	Middle frontal gyrus	mPFC
14	Inferior frontal gyrus, Superior temporal gyrus	Right IFG	**29**	Middle frontal gyrus, Superior frontal gyrus	mPFC
15	Inferior frontal gyrus	Right IFG	**30**	Superior frontal gyrus	mPFC

In this study, the connections between the frontal and the temporoparietal regions were defined as the connections between channels over the mPFC (channel 27, 28, 29, 30) and channels that were more closely identified over other regions belonging the DMN, such as the left superior temporal gyrus (left STG, channel 5, 6, 7), the right superior temporal gyrus (right STG, channel 18, 19, 20), the left middle/posterior temporal gyrus (left PTG, channel 8, 10, 11, 13), the right middle/posterior temporal gyrus (right PTG, channel 21, 23, 24, 26), the left TPJ (channel 9, 12), the right TPJ (channel 22, 25).

### 2.6 Resting-state data pre-processing and analysis

**Fig. 5:**
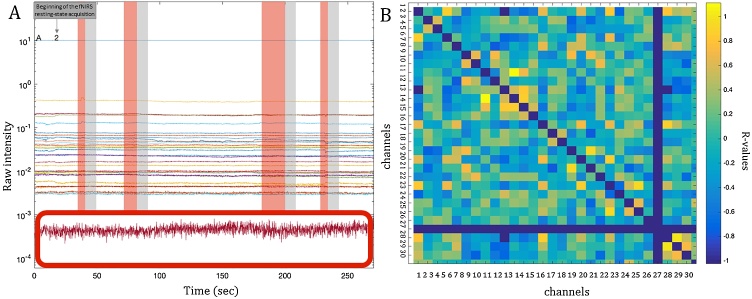
A, Representation of the fNIRS resting-state data acquired. In the lower part of the figure, a red box marks channels excluded from the analysis because of a mean intensity lower than 10^−3^ optical units. On the remaining channels, red windows mark chunks excluded based on the behavioural coding. The grey windows represent the 8 s of additional data excluded after each invalid section. B, Correlation matrix of 30 × 30 channels. Blue lines indicate channels that were excluded because of the pre-processing (the diagonal blue line indicates the correlation of the channels with themselves).

Data analysis was carried out in MATLAB (Mathworks, USA). fNIRS data were extracted for each participant from all the channels for both HbO_2_ and HHb and we excluded channels with mean intensity lower than 10^−3^ optical units, indicating bad optode-scalp coupling. This threshold was dictated by the intrinsic characteristics of the UCL-NIRS topography system and by and our own experience with this system (Fig. 5,A).

Videos of the testing session were coded offline and periods during which the toddler moved, cried, or looked at something socially engaging (e.g. the parent or the experimenter) were marked as invalid, as well as periods of time during which the parent or experimenter was talking. The behavioural coding was performed with Mangold-INTERACT software (https://www.mangold-international.com/en/). To assess inter-coder reliability, videos of five randomly chosen Recognisers and five randomly chosen Non-Recognisers participants were blindly double-coded by another researcher. We found high reliability between the two coders (k = 0.88).

Assuming 8 s to be the minimum amount of time it takes for the toddler HRF to return to baseline levels (Lloyd-Fox et al., 2010; Taga et al., 2011), 8 s of data across all the channels were excluded after each invalid section, to ensure that we were only including periods of resting state. Sections of valid data were included only if they were at least 5 consecutive seconds long (uninterrupted) (Fig. 5,A). After the behavioural coding, time series for each fNIRS channel were extracted for each participant and only participants who had at least 100 s of clean data^5^ in total, and less than 30% of the channels excluded were included in further analyses. The light attenuation values were band-pass filtered (0.01–0.08) and converted to relative concentrations of haemoglobin using the modified Beer-Lambert law (Villringer and Chance, 1997). For each participant, the correlation matrix between all the channels that survived pre-processing was calculated for both HbO_2_ and HHb, resulting in a 30 × 30 matrix of R-values (Fig. 5,B). We then applied Fisher z-transformation on the correlation matrix for further statistical analyses. Pairs of functional connections were included in the analysis only if at least half of the sample contributed data to the statistical tests (see Supplementary materials for the degrees of freedom of the t-tests between the two groups in each connection).

For each pair of channels in our analysis, we calculated if the two regions show reliable connectivity with a one sample *t*-test for the HbO_2_ and HHb signal. Then we calculated if connectivity is greater in the Recognisers than the Non-Recognisers using an independent sample t-tests in the HbO_2_ and HHb signal. For both of these, it is important to have an appropriate correction for multiple comparisons. First, we assessed for consistency between the HbO_2_ and the HHb signal, as this is an indicator of good fNIRS signal, ruling out possible artefacts of physiological noise (Tachtsidis and Scholkmann, 2016). Secondly, we assessed difference in connectivity between the two groups on ROIs (as defined in Section 2.5), and significant results were corrected for multiple comparisons using the False Discovery Rate (FDR) method (Benjamini and Hochberg, 1995; Singh and Dan, 2006).

## 3 Results

Out of the 43 toddlers that contributed data to the RSFC analyses, 18 were classified as Recognisers and 22 as Non-Recognisers. Only 3 participants were classified as Ambiguous, and given the small size of this group, we focused our analysis only on toddlers who clearly fell into the Recogniser and Non-Recogniser categories. There were no significant differences between the two groups in parameters that could potentially affect resting-state, such as age (Recognisers: mean = 555.44 days, range = 534–571 days; Non-Recognisers: mean = 550.54 days, range = 527–568 days; t (38) = 1.18, p = 0.24) and total length of the dataset after cleaning (mean ± SD Recognisers = 164.65 ± 69.23 s, mean ± SD Non-Recognisers = 187.03 ± 61.61 s; t (38) = 1.08, p = 0.28).

**Fig. 6:**
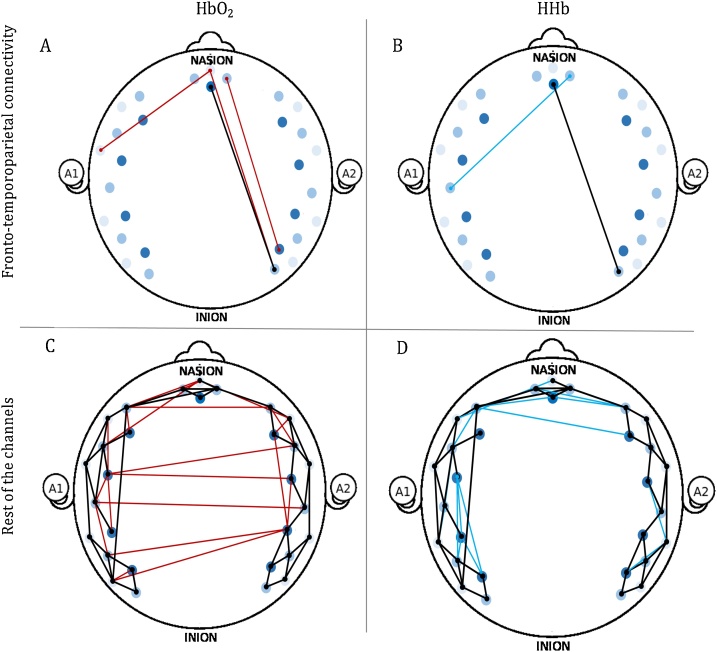
Graphical representation of the one sample t-tests in the whole sample within the fronto-temporoparietal regions and within the rest of the channels. HbO_2_ is plotted in red, HHb is plotted in blue. A, fronto-temporoparietal connections, HbO_2_ signal; B, fronto-temporoparietal connections, HHb signal; C, Rest of the channels, HbO_2_ signal; D, Rest of the channels, HHb signal. Connections that are significantly different from zero both in the HbO_2_ and the HHb signal are plotted in black.

Previous research with adults has explored the relationship between HbO_2_ and HHb in fNIRS resting-state data, and revealed a comparable pattern of spontaneous fluctuation of the two signals (Lu et al., 2010; Mesquita et al., 2010; Niu and He, 2014; Niu et al., 2011; White et al., 2009), with less connections in the HHb signal than in the HbO_2_ signal (Lu et al., 2010). Therefore, prior to any further analyses, we tested the consistency of the connectivity patterns between the HbO_2_ and HHb signal, by performing one sample t-tests on the Fisher-transformed correlation coefficients on both signals in the whole sample. Fig. 6 shows fronto-temporoparietal connections that were significantly different from zero in the whole sample, for both HbO_2_ and HHb. Significant functional connections within the rest of the channels were also plotted to assess consistency between HbO_2_ and HHb not only limited to the fronto-temporoparietal areas but also between the rest of the channels.

The functional connections significantly different from zero in the HbO_2_ and in the HHb signal revealed comparable patterns in terms of location and number of connections between the two signals. In fact, 1 out of 2 connections in the HHb signal overlap with those in the HbO_2_ signal within the fronto-temporoparietal regions, and 44 out of 56 connections in the HHb signal overlap with those in the HbO_2_ signal within the rest of the channels.

**Fig. 7:**
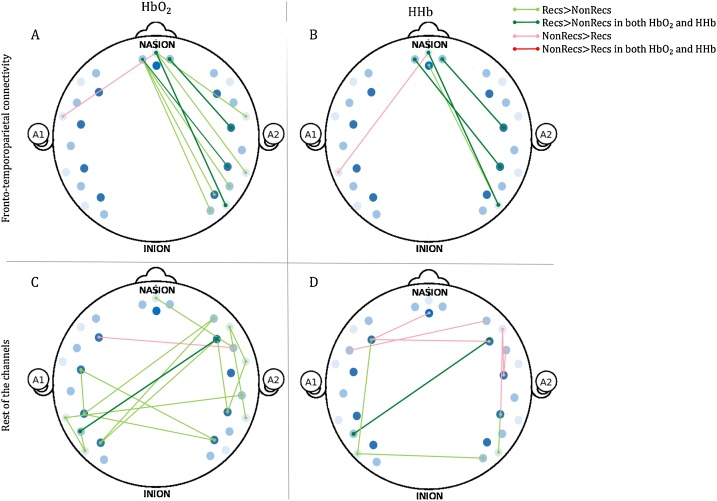
Graphical representation of the differences in fronto-temporoparietal connectivity between Recognisers and Non-Recognisers. A, fronto-temporoparietal connections, HbO_2_ signal; B, fronto-temporoparietal connections, HHb signal; C, Rest of the channels, HbO_2_ signal; D, Rest of the channels, HHb signal.

In order to test our hypothesis that there should be greater functional connectivity between the fronto-temporoparietal regions in toddlers who exhibited self-recognition compared to those who did not, we compared the Fisher-transformed correlation coefficients of Recognisers and Non-Recognisers using independent sample t-tests. Driven by our hypothesis, the main interest was to compare Recognisers and Non-Recognisers on the fronto-temporoparietal connections – as a proxy for the DMN. To assess that differences in functional connectivity between the two groups are specific of regions belonging to the DMN, we compared functional connections between Recognisers and Non-Recognisers within the rest of the channels as well. Fig. 7 shows connections that were significantly different between the two groups within both the HbO_2_ and the HHb signals (p < 0.05, uncorrected). All the pairs of functional connections were analysed, as long as more than half of the sample for whom data was collected over those channels contributed to the statistical tests. See Supplementary materials for the degrees of freedom of the *t*-test between the two groups in each connection and for the difference in connectivity between Recognisers and Non-Recognisers extended to the 44-channel configuration from which we recorded data in 23 participants.

Within the fronto-temporoparietal region, 8 connections were stronger in the Recognisers than in Non-Recognisers in the HbO_2_ signal, and 4 in the HHb signal. 3 out 4 connections that are stronger in the Recognisers than in the Non-Recognisers in the HHb signal overlap with those in the HbO_2_ signal. Only 1 connection was stronger in the Non-Recognisers than in Recognisers in the HbO_2_ signal, and 1 in the HHb signal, but they did not overlap. To increase the power of our analysis and reduce the number of multiple comparisons, we performed the independent sample t-tests on the ROIs (as defined in Section 2.5). To ensure statistical reliability, significant results were corrected for multiple comparisons using the FDR method (Benjamini and Hochberg, 1995; Singh and Dan, 2006). In the HbO_2_ signal, Recognisers showed stronger connectivity between the mPFC and the right TPJ, t (38) = 2.67, p = 0.01, and right PTG, t (38) = 3.82, p < 0.001, than the Non-Recognisers. Both these connections survived the FDR correction for multiple comparisons (p = 0.04 and p = 0.001, respectively). In the HHb signal, there was no significant difference between Recognisers and Non-Recognisers.

Within the rest of the channels, 13 connections were stronger in the Recognisers than in Non-Recognisers in the HbO_2_ signal, and 4 in the HHb signal. 1 out 4 connections that are stronger in the Recognisers than in the Non-Recognisers in the HHb signal overlap with those in the HbO_2_ signal. Only 1 connection was stronger in the Non-Recognisers than in Recognisers in the HbO_2_ signal, and 6 in the HHb signal, but they did not overlap. To increase the power of our analysis, we assessed the difference in functional connectivity between ROIs within and outside the DMN. The DMN network was composed of connections between the mPFC left and right STG, left and right left middle/posterior temporal gyrus and left and right TPJ (as specified in Section 2.5). Connections outside the DMN were tested inter-hemispheric between IFG, STG and middle/posterior temporal gyrus. In the HbO_2_ signal, a repeated measures ANOVA with network (DMN vs outside the DMN) and hemisphere (right vs. left) as a within-subjects factors and group as a between-subjects factor (Recognisers vs Non-Recognisers), showed a non-significant interaction between network hemisphere and group, F(38) = 2.95, p = 0.085. We included hemisphere as an additional within-subjects factor as analysis on a channel-based showed differences in connectivity between the two groups predominantly in the right hemisphere. As the repeated measures ANOVA showed a trend towards significance, we performed a repeated measures ANOVA with network (DMN vs outside the DMN) as a within-subject factors and group as a between-subjects factor (Recognisers vs Non-Recognisers) per each hemisphere. There was a significant interaction between network and group in the right hemisphere, F(38) = 7.87, p = 0.008, but not in the left hemisphere, F(38) = 0.198, p = 0.659. Finally, we followed up these ANOVAs with independent t-tests between Recognisers and Non-Recognisers. The Recognisers showed significantly stronger connectivity than Non-Recognisers in regions belonging the DMN on the right hemisphere, t(38) = 3.14, p = 0.003 but not on the left hemisphere t(38) = 0.038, p = 0.201. There was also marginally significant greater connectivity in the Recognisers compared to Non-Recognisers outside the DMN in the right hemisphere, t(38) = 1.99, p = 0.075 and not in the left t(38) = 0.054, p = 0.297.

We followed the same pipeline of analysis for the HHb signal. A repeated measures ANOVA with network (DMN vs outside the DMN) and hemisphere (right vs. left) as a within-subjects factors and group as a between-subjects factor (Recognisers vs Non-Recognisers), showed a non-significant interaction between network, hemisphere and group, F(38) = 1.10, p = 0.029. The follow-up ANOVA by hemisphere showed a nearly significant effect in the right hemisphere, F(38) = 3.42, p = 0.07, and a significant effect in the left hemisphere F(38) = 14.41, p = 0.001. The Recognisers showed nearly significant stronger connectivity than Non-Recognisers in regions belonging the DMN on the right hemisphere, t(38) = 1.82, p = 0.073 but not on the left hemisphere t(38) = 1.41, p = 0.166. Non-Recognisers showed significantly greater connectivity outside the DMN the left hemisphere, t(38)=-2.62, p = 0.012, but not in the right t(38)=-0.197, p=0.845.

## 4 Discussion

While investigating the ontogeny of self-awareness remains challenging (Zahavi and Roepstorff, 2011), one important way of moving this field of study forward is to develop new indices of self-awareness, that can complement and add validity to the mirror self-recognition task. While it has been claimed that the MSR test indexes toddlers’ emerging self-awareness (Gallup et al., 2016), beyond mere physical self-recognition, there is still no general agreement that this is the case. To date, our confidence in the MSR test as a measure of emerging self-awareness is limited by both a lack of alternative age-appropriate self-related tasks against which performance on the MSR can be compared, and alternative explanations for success on the MSR test which do not involve self-related processing (e.g. Heyes, 1994). In the current study, we addressed this challenge by asking whether a functional network of brain regions –the so-called default mode network which is commonly thought to be involved in psychological or cognitive self-related processing in adults – is associated with MSR in infancy. Specifically, we hypothesized that if the MSR task reflects self-related processing (Bischof-Köhler, 2012; Howe et al., 1993) and not merely recognition of the physical self (Heyes, 1994), or a matching of seen and felt movements (Mitchell, 1993a,b), then resting-state activity in regions comprising the DMN – thought to reflect self-related processing in adults - might be expected to be higher in those toddlers who do recognize themselves in the mirror, compared with those who do not show evidence of mirror self-recognition. Our findings support this hypothesis and suggest that fronto-temporoparietal connectivity is associated with self-recognition in infancy, suggesting that this measure might be considered as a possible neural marker for the development of the sense of self in early development.

While we cannot claim that this fronto-temporoparietal connectivity reflects the entire DMN, this increased connectivity in recognizers is consistent with previous adult reports of a link between frontal and temporoparietal areas and the sense of self (Davey et al., 2016; Molnar-Szakacs and Uddin, 2013; Philippi, 2012). Indeed, our hypothesis was generated based on the known role of the DMN in self-related processing (Davey et al., 2016; Golland et al., 2008; Molnar-Szakacs and Uddin, 2013; Raichle, 2015; Sporns, 2010), and the hypothesized association between MSR and self-awareness (Asendorpf et al., 1996; Bischof-Köhler, 2012; Lewis and Ramsay, 2004; Rochat, 2003; Savanah, 2013; Suddendorf and Butler, 2013). Thus, the observed pattern of stronger fronto-temporoparietal connectivity exhibited by toddlers who demonstrated mirror self-recognition at 18 months of age, might plausibly be supported by an advanced integration in a network of core areas for self-processing. The functional connections between these areas at rest, present to a greater extent in those toddlers who exhibited self-recognition, may plausibly underlie an ongoing process of monitoring self-relevant internal signals and thoughts during the absence of any specific cognitive and social stimulation. However, while we found a relationship between functional connectivity in this self-related processing network of brain regions and mirror self-recognition, we do not know what role activation in these regions plays in the ability to exhibit MSR. One possibility is that some minimal level of functional connectivity between regions of the DMN is required for self-recognition because the DMN supports self-related processing, and MSR reflects self-related processing. While our data is consistent with such an interpretation, we also recognize that without longitudinal measurements, we cannot claim a causal relationship between RSFC in the DMN and success on the MSR task. It is also possible that those infants who recognize themselves in the mirror at 18 months, had greater RSFC within this same network of brain regions at a much younger age, and increased integration between these regions facilitates a range of abilities, only one of which is self-recognition and self-awareness. Thus, while our data are consistent with the purported relationship between mirror self-recognition and self-awareness supported by regions comprising the DMN, longitudinal work would be needed to explore whether development of the DMN is causally related to MSR.

Our finding of a possible role for the fronto-temporoparietal connections in the emergence of self-awareness is broadly consistent with the only previous study which has investigated the neural basis of MSR in toddlers. In that study, Lewis and Carmody (2008) found that toddlers who recognized themselves in the mirror showed greater maturation of the left TPJ, a region involved in the DMN. In the current study however, the vast majority of the fronto-temporoparietal connections that were stronger in Recognizers than in Non-Recognizers were observed in the right hemisphere, a tendency which has also been reported in previous adult studies (Keenan et al., 2001; Kircher et al., 2001; Molnar-Szakacs and Uddin, 2013; Platek et al., 2004, 2006; Sugiura et al., 2005). Moreover, a recent study that aimed to identify structural brain correlates of MSR in chimpanzees, reported increased right hemisphere fronto-parietal white matter connectivity in chimpanzees who passed the MSR task (Hecht et al., 2017). While these previous studies analysed structural connectivity, our study provides additional evidence for the importance of this network of brain regions by demonstrating a relationship between functional connectivity in these areas, and MSR. However, as evidence of a possible lateralisation of the neural correlates of emerging self-awareness is still very limited, this hemisphere-specific effect would benefit from confirmatory replication.

An alternative interpretation of our findings should acknowledge the possibility that the functional connectivity we observe in the Recognisers reflects a generally more mature brain, which also gives rise to a more mature level of self-awareness (Fair et al., 2008; Gao et al., 2009, 2014; Nathan Spreng and Schacter, 2012). Recognizers also showed increased brain connectivity on channels outside the DMN. When data are averaged across ROIs, the greater connectivity displayed by the Recognisers seems to be specific of the DMN network, both in the HbO_2_ and in the HHb signal, which might suggest that differences in connectivity between the two groups were specific to regions belonging to the DMN. This would be consistent with previous studies which have found differences in empathy (Bischof-Köhler, 2012) and white matter maturation (Lewis and Carmody, 2008) related to the development of the sense of self, even when controlling for age. However, the only way to know whether infants who show MSR have specifically increased RSFC in the DMN, or have broadly more mature brains, would be to acquire MRI images to assess structural and functional connectivity and cortical thickness as an index of maturation of the brain. Nevertheless, even if these functional connectivity differences between Recognizers and Non-Recognisers were not specific to the DMN, it may still be that DMN maturity plays a specific role in promoting self-recognition.

Another limitation of this study is the fact that we were unable to investigate connectivity in the entire DMN, as we could only measure from the surface of the cortex that is accessible by fNIRS. However, we benefited from the excellent suitability of fNIRS for the acquisition of resting-state data from toddlers during quiet wakefulness, which most closely approximates the recording conditions under which resting-state data is typically acquired in adults. As a result, our data were likely less affected by motion artefacts than it would have been had we used fMRI. The high consistency between the HbO_2_ and the HHb signals is in line with fNIRS resting-state data acquired by previous studies, suggesting reliability of the data acquired (Lu et al., 2010; Mesquita et al., 2010; Niu and He, 2014; Niu et al., 2011; White et al., 2009).

With this study, we have shown that fronto-temporoparietal functional connectivity, considered here as a possible proxy for the DMN, may play a role in emerging self-awareness. Given the considerable volume of data suggesting that the DMN is involved in self-related processing, we consider the observed positive relationship between fronto-temporoparietal functional connectivity and MSR to be consistent with the hypothesis that MSR reflects self-awareness. If this is the case, future research should find that the same brain regions are recruited during active tasks of self-processing in infants, if it were possible to collect this data during the process of mirror self-recognition. In fact, several adult studies have observed that two core regions of the DMN, the mPFC and the TPJ, are recruited in self-processing tasks (Davey et al., 2016; Kaplan et al., 2008; Kelley et al., 2002; Kircher et al., 2000; Uddin et al., 2005). Activation of the mPFC has been observed in tasks where participants looked at their own faces or listened to their own voices (Kampe et al., 2003; Platek et al., 2006; Staffen et al., 2006; Sugiura et al., 2008, 2012), but also related to several forms of self-reflection (Jenkins and Mitchell, 2011). The TPJ has been found to be particularly engaged in self-other distinction (Decety and Lamm, 2007; Lamm et al., 2016; Santiesteban et al., 2015; Steinbeis, 2016). If our interpretation of the current data as providing support for the hypothesis that MSR entails self-awareness (Asendorpf et al., 1996; Rochat, 2003; Rochat and Zahavi, 2011; Suddendorf and Butler, 2013; Zahavi et al., 2004), then we should expect to see recruitment of these regions also during active tasks of self-awareness in infants.

## Declaration of Competing Interest

None.

## Footnotes

1 In order to infer more precisely the channel locations, we co-registered the fNIRS array on MRI images (see Section 2.5 for more details).

2 Before the beginning of the study, participant’s head measurements (circumference, the distance between ears over the forehead, distance between nasion to inion, distance between ears measured over the top of the head) were taken to select the most appropriate cap size.

3 Mind-wandering can affect resting-state functional connectivity differently as shown by Chou and colleagues (Chou et al., 2017)

4 Based on the International 10-20 EEG placement system.

5 A recent study on children showed that as little as 1 minute of resting-state fNIRS recording is sufficient to obtain accurate functional connectivity estimation (Wang et al., 2017).
