# Supplementary material for: Fronto-temporoparietal connectivity and self-awareness in 18-month-olds: A resting state fNIRS study
Source: Dev Cogn Neurosci. 2019 Jun 22;38:100676. doi: 10.1016/j.dcn.2019.100676 (PMC6969340; doi:10.1016/j.dcn.2019.100676)
Supplement: Supplementary file 1 [file mmc1.docx]

**Supplementary Materials**

Here we report fronto-temporoparietal connections that significantly different from zero in the whole sample in the 44-channel configuration, for both HbO_2_ and HHb. Significant functional connections within the rest of the channels were also plotted to assess consistency between HbO_2_ and HHb not only limited to the fronto-temporoparietal areas but also between the rest of the channels.

**Supplementary Figure 1** Graphical representation of the one sample t-tests in the whole sample within the fronto-temporoparietal regions and within the rest of the channels. HbO_2_ is plotted in red, HHb is plotted in blue. A, fronto-temporoparietal connections, HbO_2_ signal; B, fronto-temporoparietal connections, HHb signal; C, Rest of the channels, HbO_2_ signal; D, Rest of the channels, HHb signal. Connections that are significantly different from zero both in the HbO_2_ and the HHb signal are plotted in black.

1 out of 2 connections that are stronger in the Recognisers than in the Non-Recognisers in the HHb signal overlap with those in the HbO_2_ signal within the fronto-temporoparietal regions, and 58 out of 78 connections in the HHb signal overlap with those in the HbO_2_ signal within the rest of the channels.

To test our hypothesis that there should be greater functional connectivity between the fronto-temporoparietal regions in toddlers who exhibited self-recognition compared to those who did not, we compared the Fisher-transformed correlation coefficients of Recognisers and Non-Recognisers using independent sample t-tests. We report here connections that were significantly different between the two groups within both the HbO_2_ and the HHb signals (p<0.05, uncorrected). Supplementary Figure 2 shows connections that were significantly different between the two groups within both the HbO_2_ and the HHb signals (p<0.05, uncorrected).

D

C

**Supplementary Figure 2** Graphical representation of the differences in connectivity within the rest of the channels between Recognisers and Non-Recognisers. A, HbO_2_ signal; B, HHb signal. A, fronto-temporoparietal connections, HbO_2_ signal; B, fronto-temporoparietal connections, HHb signal; C, Rest of the channels, HbO_2_ signal; D, Rest of the channels, HHb signal.

Within the fronto-temporoparietal region, 13 connections were stronger in the Recognisers than in Non-Recognisers in the HbO_2_ signal, and 7 in the HHb signal. 5 out 7 connections in the HHb signal overlap with those in the HbO_2_ signal. Only 1 connection was stronger in the Non-Recognisers than in Recognisers in the HbO_2_ signal, and 1 in the HHb signal, but they did not overlap. (See Supplementary Table 1 and 2 for the degrees of freedom of the t-test between the two groups in each connection). Within the rest of the channels, 35 connections were stronger in the Recognisers than in Non-Recognisers in the HbO_2_ signal, and 9 in the HHb signal. 4 out 9 connections that are stronger in the Recognisers than in the Non-Recognisers in the HHb signal overlap with those in the HbO_2_ signal. Only 7 connection was stronger in the Non-Recognisers than in Recognisers in the HbO_2_ signal, and 9 in the HHb signal. 1 of which overlap with the one in the HbO_2_ signal.

Here we report the degrees of freedom of the t-tests between the Recognisers and the Non-Recognisers for each connection (right side of the table). Degrees of freedom of the connections that showed a significant difference between Recognisers and Non-Recognisers are marked in black. We report also p-values of the pairs of channels that showed a significant difference between Recognisers and Non-Recognisers (right side of the table).

Supplementary Table 1 reports degrees of freedom and p-values related to the t-tests performed on the HbO2 signal, and Supplementary Table 2 reports degrees of freedom and p-values related to the t-tests performed on the HHb signal.

**Degrees of freedom**

channels

**p-values**

channels

|  | **1** | **2** | **3** | **4** | **5** | **6** | **7** | **8** | **9** | **10** | **11** | **12** | **13** | **14** | **15** | **16** | **17** | **18** | **19** | **20** | **21** | **22** | **23** | **24** | **25** | **26** | **27** | **28** | **29** | **30** | **31** | **32** | **33** | **34** | **35** | **36** | **37** | **38** | **39** | **40** | **41** | **42** | **43** | **44** |
| --- | --- | --- | --- | --- | --- | --- | --- | --- | --- | --- | --- | --- | --- | --- | --- | --- | --- | --- | --- | --- | --- | --- | --- | --- | --- | --- | --- | --- | --- | --- | --- | --- | --- | --- | --- | --- | --- | --- | --- | --- | --- | --- | --- | --- |
| **1** |  | 36 | 36 | 35 | 35 | 35 | 34 | 36 | 35 | 35 | 33 | 30 | 29 | 36 | 36 | 36 | 35 | 36 | 35 | 34 | 35 | 34 | 36 | 35 | 34 | 32 | 33 | 33 | 35 | 33 | 18 | 17 | 18 | 17 | 17 | 15 | 16 | 18 | 17 | 17 | 15 | 15 | 14 | 17 |
| **2** |  |  | 38 | 35 | 35 | 36 | 35 | 38 | 35 | 35 | 35 | 32 | 31 | 38 | 38 | 38 | 37 | 38 | 36 | 36 | 37 | 36 | 38 | 37 | 36 | 34 | 35 | 35 | 36 | 35 | 20 | 19 | 19 | 19 | 19 | 17 | 18 | 20 | 17 | 19 | 16 | 16 | 15 | 19 |
| **3** |  |  |  | 35 | 35 | 36 | 35 | 38 | 35 | 35 | 35 | 32 | 31 | 38 | 38 | 38 | 37 | 38 | 36 | 36 | 37 | 36 | 38 | 37 | 36 | 34 | 35 | 35 | 36 | 35 | 20 | 19 | 19 | 19 | 19 | 17 | 18 | 20 | 17 | 19 | 16 | 16 | 15 | 19 |
| **4** |  |  |  |  | 35 | 34 | 34 | 35 | 34 | 34 | 33 | 29 | 29 | 35 | 35 | 35 | 34 | 35 | 34 | 34 | 34 | 33 | 35 | 34 | 34 | 32 | 32 | 33 | 34 | 33 | 18 | 17 | 18 | 17 | 17 | 15 | 16 | 18 | 17 | 17 | 15 | 15 | 14 | 17 |
| **5** |  |  |  |  |  | 34 | 34 | 35 | 34 | 34 | 33 | 29 | 29 | 35 | 35 | 35 | 34 | 35 | 34 | 34 | 34 | 33 | 35 | 34 | 34 | 32 | 32 | 33 | 34 | 33 | 18 | 17 | 18 | 17 | 17 | 15 | 16 | 18 | 17 | 17 | 15 | 15 | 14 | 17 |
| **6** |  |  |  |  |  |  | 35 | 36 | 35 | 34 | 33 | 31 | 30 | 36 | 36 | 36 | 35 | 36 | 35 | 34 | 35 | 34 | 36 | 35 | 35 | 33 | 33 | 33 | 35 | 33 | 19 | 18 | 19 | 18 | 18 | 16 | 17 | 19 | 17 | 18 | 16 | 16 | 14 | 18 |
| **7** |  |  |  |  |  |  |  | 35 | 34 | 33 | 33 | 30 | 30 | 35 | 35 | 35 | 34 | 35 | 34 | 34 | 34 | 33 | 35 | 34 | 35 | 33 | 32 | 33 | 34 | 33 | 19 | 18 | 19 | 18 | 18 | 16 | 17 | 19 | 17 | 18 | 16 | 16 | 14 | 18 |
| **8** |  |  |  |  |  |  |  |  | 35 | 35 | 35 | 32 | 31 | 38 | 38 | 38 | 37 | 38 | 36 | 36 | 37 | 36 | 38 | 37 | 36 | 34 | 35 | 35 | 36 | 35 | 20 | 19 | 19 | 19 | 19 | 17 | 18 | 20 | 17 | 19 | 16 | 16 | 15 | 19 |
| **9** |  |  |  |  |  |  |  |  |  | 34 | 32 | 30 | 29 | 35 | 35 | 35 | 34 | 35 | 34 | 33 | 34 | 33 | 35 | 34 | 34 | 32 | 32 | 32 | 34 | 32 | 18 | 17 | 18 | 17 | 17 | 15 | 16 | 18 | 17 | 17 | 15 | 15 | 14 | 17 |
| **10** |  |  |  |  |  |  |  |  |  |  | 32 | 29 | 29 | 35 | 35 | 35 | 34 | 35 | 34 | 33 | 34 | 33 | 35 | 34 | 33 | 31 | 32 | 32 | 34 | 32 | 17 | 16 | 17 | 16 | 16 | 14 | 15 | 17 | 16 | 16 | 14 | 14 | 14 | 16 |
| **11** |  |  |  |  |  |  |  |  |  |  |  | 30 | 30 | 35 | 35 | 35 | 35 | 35 | 34 | 35 | 35 | 34 | 35 | 34 | 34 | 32 | 32 | 33 | 33 | 33 | 18 | 17 | 17 | 17 | 17 | 15 | 17 | 18 | 15 | 18 | 15 | 15 | 14 | 17 |
| **12** |  |  |  |  |  |  |  |  |  |  |  |  | 30 | 32 | 32 | 32 | 32 | 32 | 31 | 31 | 32 | 31 | 32 | 31 | 31 | 29 | 29 | 29 | 30 | 29 | 18 | 17 | 17 | 17 | 18 | 16 | 17 | 18 | 15 | 18 | 15 | 15 | 14 | 17 |
| **13** |  |  |  |  |  |  |  |  |  |  |  |  |  | 31 | 31 | 31 | 31 | 31 | 30 | 31 | 31 | 30 | 31 | 30 | 31 | 29 | 28 | 29 | 29 | 29 | 18 | 17 | 17 | 17 | 17 | 15 | 17 | 18 | 15 | 18 | 15 | 15 | 14 | 17 |
| **14** |  |  |  |  |  |  |  |  |  |  |  |  |  |  | 38 | 38 | 37 | 38 | 36 | 36 | 37 | 36 | 38 | 37 | 36 | 34 | 35 | 35 | 36 | 35 | 20 | 19 | 19 | 19 | 19 | 17 | 18 | 20 | 17 | 19 | 16 | 16 | 15 | 19 |
| **15** |  |  |  |  |  |  |  |  |  |  |  |  |  |  |  | 38 | 37 | 38 | 36 | 36 | 37 | 36 | 38 | 37 | 36 | 34 | 35 | 35 | 36 | 35 | 20 | 19 | 19 | 19 | 19 | 17 | 18 | 20 | 17 | 19 | 16 | 16 | 15 | 19 |
| **16** |  |  |  |  |  |  |  |  |  |  |  |  |  |  |  |  | 37 | 38 | 36 | 36 | 37 | 36 | 38 | 37 | 36 | 34 | 35 | 35 | 36 | 35 | 20 | 19 | 19 | 19 | 19 | 17 | 18 | 20 | 17 | 19 | 16 | 16 | 15 | 19 |
| **17** |  |  |  |  |  |  |  |  |  |  |  |  |  |  |  |  |  | 37 | 36 | 36 | 37 | 36 | 37 | 36 | 35 | 33 | 34 | 34 | 35 | 34 | 19 | 18 | 18 | 18 | 18 | 16 | 18 | 19 | 16 | 19 | 16 | 16 | 15 | 18 |
| **18** |  |  |  |  |  |  |  |  |  |  |  |  |  |  |  |  |  |  | 36 | 36 | 37 | 36 | 38 | 37 | 36 | 34 | 35 | 35 | 36 | 35 | 20 | 19 | 19 | 19 | 19 | 17 | 18 | 20 | 17 | 19 | 16 | 16 | 15 | 19 |
| **19** |  |  |  |  |  |  |  |  |  |  |  |  |  |  |  |  |  |  |  | 35 | 36 | 35 | 36 | 35 | 34 | 32 | 33 | 33 | 35 | 33 | 18 | 17 | 18 | 17 | 17 | 15 | 17 | 18 | 16 | 18 | 16 | 16 | 14 | 17 |
| **20** |  |  |  |  |  |  |  |  |  |  |  |  |  |  |  |  |  |  |  |  | 36 | 35 | 36 | 35 | 35 | 33 | 33 | 34 | 34 | 34 | 19 | 18 | 18 | 18 | 18 | 16 | 18 | 19 | 16 | 19 | 16 | 16 | 15 | 18 |
| **21** |  |  |  |  |  |  |  |  |  |  |  |  |  |  |  |  |  |  |  |  |  | 36 | 37 | 36 | 35 | 33 | 34 | 34 | 35 | 34 | 19 | 18 | 18 | 18 | 18 | 16 | 18 | 19 | 16 | 19 | 16 | 16 | 15 | 18 |
| **22** |  |  |  |  |  |  |  |  |  |  |  |  |  |  |  |  |  |  |  |  |  |  | 36 | 35 | 34 | 32 | 33 | 33 | 34 | 33 | 18 | 17 | 17 | 17 | 17 | 15 | 17 | 18 | 15 | 18 | 15 | 15 | 14 | 17 |
| **23** |  |  |  |  |  |  |  |  |  |  |  |  |  |  |  |  |  |  |  |  |  |  |  | 37 | 36 | 34 | 35 | 35 | 36 | 35 | 20 | 19 | 19 | 19 | 19 | 17 | 18 | 20 | 17 | 19 | 16 | 16 | 15 | 19 |
| **24** |  |  |  |  |  |  |  |  |  |  |  |  |  |  |  |  |  |  |  |  |  |  |  |  | 35 | 34 | 35 | 34 | 35 | 35 | 20 | 19 | 19 | 19 | 19 | 17 | 18 | 20 | 17 | 19 | 16 | 16 | 15 | 19 |
| **25** |  |  |  |  |  |  |  |  |  |  |  |  |  |  |  |  |  |  |  |  |  |  |  |  |  | 34 | 33 | 34 | 34 | 34 | 20 | 19 | 19 | 19 | 19 | 17 | 18 | 20 | 17 | 19 | 16 | 16 | 15 | 19 |
| **26** |  |  |  |  |  |  |  |  |  |  |  |  |  |  |  |  |  |  |  |  |  |  |  |  |  |  | 32 | 32 | 32 | 33 | 20 | 19 | 19 | 19 | 19 | 17 | 18 | 20 | 17 | 19 | 16 | 16 | 15 | 19 |
| **27** |  |  |  |  | .029 |  |  |  |  |  |  |  |  |  |  |  |  | .026 |  |  | .042 |  |  | .005 |  |  |  | 32 | 34 | 33 | 18 | 17 | 17 | 17 | 17 | 15 | 16 | 18 | 15 | 17 | 14 | 14 | 14 | 17 |
| **28** |  |  |  |  |  |  |  |  |  |  |  |  |  |  |  |  |  |  |  |  |  | .015 | .014 |  | .015 | .017 |  |  | 33 | 34 | 19 | 18 | 18 | 18 | 18 | 16 | 17 | 19 | 16 | 18 | 15 | 15 | 14 | 18 |
| **29** |  |  |  |  |  |  |  |  |  |  |  |  |  |  |  |  |  |  | .026 |  |  |  |  |  |  |  |  |  |  | 33 | 18 | 17 | 18 | 17 | 17 | 15 | 16 | 18 | 16 | 17 | 15 | 15 | 14 | 17 |
| **30** |  |  |  |  |  |  |  |  |  |  |  |  |  |  |  |  |  |  |  |  |  |  |  |  |  |  |  |  |  |  | 20 | 19 | 19 | 19 | 19 | 17 | 18 | 20 | 17 | 19 | 16 | 16 | 15 | 19 |
| **31** |  |  |  |  |  |  |  |  |  |  |  |  |  |  |  |  |  |  |  |  |  |  |  |  |  |  |  |  |  |  |  | 19 | 19 | 19 | 19 | 17 | 18 | 20 | 17 | 19 | 16 | 16 | 15 | 19 |
| **32** |  |  |  |  |  |  |  |  |  |  |  |  |  |  |  |  |  |  |  |  |  |  |  |  |  |  |  |  |  |  |  |  | 18 | 19 | 18 | 16 | 17 | 19 | 17 | 18 | 15 | 15 | 14 | 18 |
| **33** |  |  |  |  |  |  |  |  |  |  |  |  |  |  |  |  |  |  |  |  |  |  |  |  |  |  |  |  |  |  |  |  |  | 18 | 18 | 16 | 17 | 19 | 17 | 18 | 16 | 16 | 14 | 18 |
| **34** |  |  |  |  |  |  |  |  |  |  |  |  |  |  |  |  |  |  |  |  |  |  |  |  |  |  |  | .048 |  | .016 |  |  |  |  | 18 | 16 | 17 | 19 | 17 | 18 | 15 | 15 | 14 | 18 |
| **35** |  |  |  |  |  |  |  |  |  |  |  |  |  |  |  |  |  |  |  |  |  |  |  |  |  |  |  |  |  |  |  |  |  |  |  | 17 | 17 | 19 | 16 | 18 | 15 | 15 | 14 | 18 |
| **36** |  |  |  |  |  |  |  |  |  |  |  |  |  |  |  |  |  |  |  |  |  |  |  |  |  |  |  |  |  |  |  |  |  |  |  |  | 16 | 17 | 14 | 16 | 14 | 14 | 14 | 16 |
| **37** |  |  |  |  |  |  |  |  |  |  |  |  |  |  |  |  |  |  |  |  |  |  |  |  |  |  |  |  |  |  |  |  |  |  |  |  |  | 18 | 15 | 18 | 16 | 16 | 15 | 17 |
| **38** |  |  |  |  |  |  |  |  |  |  |  |  |  |  |  |  |  |  |  |  |  |  |  |  |  |  |  |  |  |  |  |  |  |  |  |  |  |  | 17 | 19 | 16 | 16 | 15 | 19 |
| **39** |  |  |  |  |  |  |  |  |  |  |  |  |  |  |  |  |  |  |  |  |  |  |  |  |  |  |  |  |  |  |  |  |  |  |  |  |  |  |  | 16 | 14 | 14 | 14 | 16 |
| **40** |  |  |  |  |  |  |  |  |  |  |  |  |  |  |  |  |  |  |  |  |  |  |  |  |  |  |  | .022 | .031 |  |  |  |  |  |  |  |  |  |  |  | 16 | 16 | 15 | 18 |
| **41** |  |  |  |  |  |  |  |  |  |  |  |  |  |  |  |  |  |  |  |  |  |  |  |  |  |  |  |  |  |  |  |  |  |  |  |  |  |  |  |  |  | 16 | 14 | 16 |
| **42** |  |  |  |  |  |  |  |  |  |  |  |  |  |  |  |  |  |  |  |  |  |  |  |  |  |  |  |  |  |  |  |  |  |  |  |  |  |  |  |  |  |  | 14 | 16 |
| **43** |  |  |  |  |  |  |  |  |  |  |  |  |  |  |  |  |  |  |  |  |  |  |  |  |  |  |  |  | .031 |  |  |  |  |  |  |  |  |  |  |  |  |  |  | 15 |
| **44** |  |  |  |  |  |  |  |  |  |  |  |  |  |  |  |  |  |  |  |  |  |  |  |  |  |  |  |  |  |  |  |  |  |  |  |  |  |  |  |  |  |  |  |  |

**Supplementary Table 1** Degrees of freedom related to the difference between Recognisers and Non-Recognisers in each connection in the HbO_2_ signal. The additional channels of the 44-channel configuration are highlighted in grey. Pairs of channels that showed significant functional connectivity are marked in black and p-values are reported on the left side of the table.

**Degrees of freedom**

channels

channels

|  | **1** | **2** | **3** | **4** | **5** | **6** | **7** | **8** | **9** | **10** | **11** | **12** | **13** | **14** | **15** | **16** | **17** | **18** | **19** | **20** | **21** | **22** | **23** | **24** | **25** | **26** | **27** | **28** | **29** | **30** | **31** | **32** | **33** | **34** | **35** | **36** | **37** | **38** | **39** | **40** | **41** | **42** | **43** | **44** |
| --- | --- | --- | --- | --- | --- | --- | --- | --- | --- | --- | --- | --- | --- | --- | --- | --- | --- | --- | --- | --- | --- | --- | --- | --- | --- | --- | --- | --- | --- | --- | --- | --- | --- | --- | --- | --- | --- | --- | --- | --- | --- | --- | --- | --- |
| **1** |  | 36 | 36 | 35 | 35 | 35 | 34 | 36 | 35 | 35 | 33 | 30 | 29 | 36 | 36 | 36 | 35 | 36 | 35 | 34 | 35 | 34 | 36 | 35 | 34 | 32 | 33 | 33 | 35 | 33 | 18 | 17 | 18 | 17 | 17 | 15 | 16 | 18 | 17 | 17 | 15 | 15 | 14 | 17 |
| **2** |  |  | 38 | 35 | 35 | 36 | 35 | 38 | 35 | 35 | 35 | 32 | 31 | 38 | 38 | 38 | 37 | 38 | 36 | 36 | 37 | 36 | 38 | 37 | 36 | 34 | 35 | 35 | 36 | 35 | 20 | 19 | 19 | 19 | 19 | 17 | 18 | 20 | 17 | 19 | 16 | 16 | 15 | 19 |
| **3** |  |  |  | 35 | 35 | 36 | 35 | 38 | 35 | 35 | 35 | 32 | 31 | 38 | 38 | 38 | 37 | 38 | 36 | 36 | 37 | 36 | 38 | 37 | 36 | 34 | 35 | 35 | 36 | 35 | 20 | 19 | 19 | 19 | 19 | 17 | 18 | 20 | 17 | 19 | 16 | 16 | 15 | 19 |
| **4** |  |  |  |  | 35 | 34 | 34 | 35 | 34 | 34 | 33 | 29 | 29 | 35 | 35 | 35 | 34 | 35 | 34 | 34 | 34 | 33 | 35 | 34 | 34 | 32 | 32 | 33 | 34 | 33 | 18 | 17 | 18 | 17 | 17 | 15 | 16 | 18 | 17 | 17 | 15 | 15 | 14 | 17 |
| **5** |  |  |  |  |  | 34 | 34 | 35 | 34 | 34 | 33 | 29 | 29 | 35 | 35 | 35 | 34 | 35 | 34 | 34 | 34 | 33 | 35 | 34 | 34 | 32 | 32 | 33 | 34 | 33 | 18 | 17 | 18 | 17 | 17 | 15 | 16 | 18 | 17 | 17 | 15 | 15 | 14 | 17 |
| **6** |  |  |  |  |  |  | 35 | 36 | 35 | 34 | 33 | 31 | 30 | 36 | 36 | 36 | 35 | 36 | 35 | 34 | 35 | 34 | 36 | 35 | 35 | 33 | 33 | 33 | 35 | 33 | 19 | 18 | 19 | 18 | 18 | 16 | 17 | 19 | 17 | 18 | 16 | 16 | 14 | 18 |
| **7** |  |  |  |  |  |  |  | 35 | 34 | 33 | 33 | 30 | 30 | 35 | 35 | 35 | 34 | 35 | 34 | 34 | 34 | 33 | 35 | 34 | 35 | 33 | 32 | 33 | 34 | 33 | 19 | 18 | 19 | 18 | 18 | 16 | 17 | 19 | 17 | 18 | 16 | 16 | 14 | 18 |
| **8** |  |  |  |  |  |  |  |  | 35 | 35 | 35 | 32 | 31 | 38 | 38 | 38 | 37 | 38 | 36 | 36 | 37 | 36 | 38 | 37 | 36 | 34 | 35 | 35 | 36 | 35 | 20 | 19 | 19 | 19 | 19 | 17 | 18 | 20 | 17 | 19 | 16 | 16 | 15 | 19 |
| **9** |  |  |  |  |  |  |  |  |  | 34 | 32 | 30 | 29 | 35 | 35 | 35 | 34 | 35 | 34 | 33 | 34 | 33 | 35 | 34 | 34 | 32 | 32 | 32 | 34 | 32 | 18 | 17 | 18 | 17 | 17 | 15 | 16 | 18 | 17 | 17 | 15 | 15 | 14 | 17 |
| **10** |  |  |  |  |  |  |  |  |  |  | 32 | 29 | 29 | 35 | 35 | 35 | 34 | 35 | 34 | 33 | 34 | 33 | 35 | 34 | 33 | 31 | 32 | 32 | 34 | 32 | 17 | 16 | 17 | 16 | 16 | 14 | 15 | 17 | 16 | 16 | 14 | 14 | 14 | 16 |
| **11** |  |  |  |  |  |  |  |  |  |  |  | 30 | 30 | 35 | 35 | 35 | 35 | 35 | 34 | 35 | 35 | 34 | 35 | 34 | 34 | 32 | 32 | 33 | 33 | 33 | 18 | 17 | 17 | 17 | 17 | 15 | 17 | 18 | 15 | 18 | 15 | 15 | 14 | 17 |
| **12** |  |  |  |  |  |  |  |  |  |  |  |  | 30 | 32 | 32 | 32 | 32 | 32 | 31 | 31 | 32 | 31 | 32 | 31 | 31 | 29 | 29 | 29 | 30 | 29 | 18 | 17 | 17 | 17 | 18 | 16 | 17 | 18 | 15 | 18 | 15 | 15 | 14 | 17 |
| **13** |  |  |  |  |  |  |  |  |  |  |  |  |  | 31 | 31 | 31 | 31 | 31 | 30 | 31 | 31 | 30 | 31 | 30 | 31 | 29 | 28 | 29 | 29 | 29 | 18 | 17 | 17 | 17 | 17 | 15 | 17 | 18 | 15 | 18 | 15 | 15 | 14 | 17 |
| **14** |  |  |  |  |  |  |  |  |  |  |  |  |  |  | 38 | 38 | 37 | 38 | 36 | 36 | 37 | 36 | 38 | 37 | 36 | 34 | 35 | 35 | 36 | 35 | 20 | 19 | 19 | 19 | 19 | 17 | 18 | 20 | 17 | 19 | 16 | 16 | 15 | 19 |
| **15** |  |  |  |  |  |  |  |  |  |  |  |  |  |  |  | 38 | 37 | 38 | 36 | 36 | 37 | 36 | 38 | 37 | 36 | 34 | 35 | 35 | 36 | 35 | 20 | 19 | 19 | 19 | 19 | 17 | 18 | 20 | 17 | 19 | 16 | 16 | 15 | 19 |
| **16** |  |  |  |  |  |  |  |  |  |  |  |  |  |  |  |  | 37 | 38 | 36 | 36 | 37 | 36 | 38 | 37 | 36 | 34 | 35 | 35 | 36 | 35 | 20 | 19 | 19 | 19 | 19 | 17 | 18 | 20 | 17 | 19 | 16 | 16 | 15 | 19 |
| **17** |  |  |  |  |  |  |  |  |  |  |  |  |  |  |  |  |  | 37 | 36 | 36 | 37 | 36 | 37 | 36 | 35 | 33 | 34 | 34 | 35 | 34 | 19 | 18 | 18 | 18 | 18 | 16 | 18 | 19 | 16 | 19 | 16 | 16 | 15 | 18 |
| **18** |  |  |  |  |  |  |  |  |  |  |  |  |  |  |  |  |  |  | 36 | 36 | 37 | 36 | 38 | 37 | 36 | 34 | 35 | 35 | 36 | 35 | 20 | 19 | 19 | 19 | 19 | 17 | 18 | 20 | 17 | 19 | 16 | 16 | 15 | 19 |
| **19** |  |  |  |  |  |  |  |  |  |  |  |  |  |  |  |  |  |  |  | 35 | 36 | 35 | 36 | 35 | 34 | 32 | 33 | 33 | 35 | 33 | 18 | 17 | 18 | 17 | 17 | 15 | 17 | 18 | 16 | 18 | 16 | 16 | 14 | 17 |
| **20** |  |  |  |  |  |  |  |  |  |  |  |  |  |  |  |  |  |  |  |  | 36 | 35 | 36 | 35 | 35 | 33 | 33 | 34 | 34 | 34 | 19 | 18 | 18 | 18 | 18 | 16 | 18 | 19 | 16 | 19 | 16 | 16 | 15 | 18 |
| **21** |  |  |  |  |  |  |  |  |  |  |  |  |  |  |  |  |  |  |  |  |  | 36 | 37 | 36 | 35 | 33 | 34 | 34 | 35 | 34 | 19 | 18 | 18 | 18 | 18 | 16 | 18 | 19 | 16 | 19 | 16 | 16 | 15 | 18 |
| **22** |  |  |  |  |  |  |  |  |  |  |  |  |  |  |  |  |  |  |  |  |  |  | 36 | 35 | 34 | 32 | 33 | 33 | 34 | 33 | 18 | 17 | 17 | 17 | 17 | 15 | 17 | 18 | 15 | 18 | 15 | 15 | 14 | 17 |
| **23** |  |  |  |  |  |  |  |  |  |  |  |  |  |  |  |  |  |  |  |  |  |  |  | 37 | 36 | 34 | 35 | 35 | 36 | 35 | 20 | 19 | 19 | 19 | 19 | 17 | 18 | 20 | 17 | 19 | 16 | 16 | 15 | 19 |
| **24** |  |  |  |  |  |  |  |  |  |  |  |  |  |  |  |  |  |  |  |  |  |  |  |  | 35 | 34 | 35 | 34 | 35 | 35 | 20 | 19 | 19 | 19 | 19 | 17 | 18 | 20 | 17 | 19 | 16 | 16 | 15 | 19 |
| **25** |  |  |  |  |  |  |  |  |  |  |  |  |  |  |  |  |  |  |  |  |  |  |  |  |  | 34 | 33 | 34 | 34 | 34 | 20 | 19 | 19 | 19 | 19 | 17 | 18 | 20 | 17 | 19 | 16 | 16 | 15 | 19 |
| **26** |  |  |  |  |  |  |  |  |  |  |  |  |  |  |  |  |  |  |  |  |  |  |  |  |  |  | 32 | 32 | 32 | 33 | 20 | 19 | 19 | 19 | 19 | 17 | 18 | 20 | 17 | 19 | 16 | 16 | 15 | 19 |
| **27** |  |  |  |  |  |  |  | .009 |  |  |  |  |  |  |  |  |  |  |  |  |  |  |  | .037 |  |  |  | 32 | 34 | 33 | 18 | 17 | 17 | 17 | 17 | 15 | 16 | 18 | 15 | 17 | 14 | 14 | 14 | 17 |
| **28** |  |  |  |  |  |  |  |  |  |  |  |  |  |  |  |  |  |  |  |  |  | .023 |  |  |  |  |  |  | 33 | 34 | 19 | 18 | 18 | 18 | 18 | 16 | 17 | 19 | 16 | 18 | 15 | 15 | 14 | 18 |
| **29** |  |  |  |  |  |  |  |  |  |  |  |  |  |  |  |  |  |  | .008 |  |  |  |  |  |  |  |  |  |  | 33 | 18 | 17 | 18 | 17 | 17 | 15 | 16 | 18 | 16 | 17 | 15 | 15 | 14 | 17 |
| **30** |  |  |  |  |  |  |  |  |  |  |  |  |  |  |  |  |  |  |  |  |  |  |  | .020 |  |  |  |  |  |  | 20 | 19 | 19 | 19 | 19 | 17 | 18 | 20 | 17 | 19 | 16 | 16 | 15 | 19 |
| **31** |  |  |  |  |  |  |  |  |  |  |  |  |  |  |  |  |  |  |  |  |  |  |  |  |  |  |  |  |  |  |  | 19 | 19 | 19 | 19 | 17 | 18 | 20 | 17 | 19 | 16 | 16 | 15 | 19 |
| **32** |  |  |  |  |  |  |  |  |  |  |  |  |  |  |  |  |  |  |  |  |  |  |  |  |  |  |  |  |  |  |  |  | 18 | 19 | 18 | 16 | 17 | 19 | 17 | 18 | 15 | 15 | 14 | 18 |
| **33** |  |  |  |  |  |  |  |  |  |  |  |  |  |  |  |  |  |  |  |  |  |  |  |  |  |  |  |  |  |  |  |  |  | 18 | 18 | 16 | 17 | 19 | 17 | 18 | 16 | 16 | 14 | 18 |
| **34** |  |  |  |  |  |  |  |  |  |  |  |  |  |  |  |  |  |  |  |  |  |  |  |  |  |  | .048 |  |  |  |  |  |  |  | 18 | 16 | 17 | 19 | 17 | 18 | 15 | 15 | 14 | 18 |
| **35** |  |  |  |  |  |  |  |  |  |  |  |  |  |  |  |  |  |  |  |  |  |  |  |  |  |  |  |  |  |  |  |  |  |  |  | 17 | 17 | 19 | 16 | 18 | 15 | 15 | 14 | 18 |
| **36** |  |  |  |  |  |  |  |  |  |  |  |  |  |  |  |  |  |  |  |  |  |  |  |  |  |  |  |  | .030 |  |  |  |  |  |  |  | 16 | 17 | 14 | 16 | 14 | 14 | 14 | 16 |
| **37** |  |  |  |  |  |  |  |  |  |  |  |  |  |  |  |  |  |  |  |  |  |  |  |  |  |  |  |  |  |  |  |  |  |  |  |  |  | 18 | 15 | 18 | 16 | 16 | 15 | 17 |
| **38** |  |  |  |  |  |  |  |  |  |  |  |  |  |  |  |  |  |  |  |  |  |  |  |  |  |  |  |  |  |  |  |  |  |  |  |  |  |  | 17 | 19 | 16 | 16 | 15 | 19 |
| **39** |  |  |  |  |  |  |  |  |  |  |  |  |  |  |  |  |  |  |  |  |  |  |  |  |  |  |  |  |  |  |  |  |  |  |  |  |  |  |  | 16 | 14 | 14 | 14 | 16 |
| **40** |  |  |  |  |  |  |  |  |  |  |  |  |  |  |  |  |  |  |  |  |  |  |  |  |  |  |  |  | .019 |  |  |  |  |  |  |  |  |  |  |  | 16 | 16 | 15 | 18 |
| **41** |  |  |  |  |  |  |  |  |  |  |  |  |  |  |  |  |  |  |  |  |  |  |  |  |  |  |  |  |  |  |  |  |  |  |  |  |  |  |  |  |  | 16 | 14 | 16 |
| **42** |  |  |  |  |  |  |  |  |  |  |  |  |  |  |  |  |  |  |  |  |  |  |  |  |  |  |  |  |  |  |  |  |  |  |  |  |  |  |  |  |  |  | 14 | 16 |
| **43** |  |  |  |  |  |  |  |  |  |  |  |  |  |  |  |  |  |  |  |  |  |  |  |  |  |  |  |  |  |  |  |  |  |  |  |  |  |  |  |  |  |  |  | 15 |
| **44** |  |  |  |  |  |  |  |  |  |  |  |  |  |  |  |  |  |  |  |  |  |  |  |  |  |  |  |  |  |  |  |  |  |  |  |  |  |  |  |  |  |  |  |  |

**p-values**

**Supplementary Table 2** Degrees of freedom related to the difference between Recognisers and Non-Recognisers in each connection in the HHb signal. The additional channels of the 44-channel configuration are highlighted in grey. Pairs of channels that showed significant functional connectivity are marked in black and p-values are reported on the left side of the table.
